# Cocaine and Opioid-Induced Acute Liver Injury: A Rare Case Report

**DOI:** 10.7759/cureus.23630

**Published:** 2022-03-29

**Authors:** Tsering Dolkar, Abubaker M Hamad, Myat M Han, Myint B Thu, Vijay R Gayam

**Affiliations:** 1 Internal Medicine, Interfaith Medical Center, One Brooklyn Health System (OBHS), Brooklyn, USA; 2 Internal Medicine, Memorial Hospital Converse County, Douglas, USA; 3 Internal Medicine, Harlem Hospital Center, New York City, USA; 4 Gastroenterology and Hepatology, The Brooklyn Hospital Center, Brooklyn, USA

**Keywords:** elevated liver associated enzymes, acute hepatotoxicity, opioids, cocaine, drug induced liver injury

## Abstract

Drug overdose has been a public health burden in the United States. Repeated use of cocaine and heroin may increase the risk of severe acute liver failure. We present the case of a middle-aged man with no significant past medical condition except a chronic history of drug abuse who presented to our hospital after an overdose of cocaine and heroin. Patient received Narcan by paramedics and continued treatment in the emergency room (ER). Patient has exhibited multiple organ failures, such as acute liver failure, rhabdomyolysis, acute kidney injury, and acute respiratory hypoxic hypercapnic respiratory failure likely due to respiratory center depression. The patient was placed on a non-rebreather mask then a bilevel positive airway pressure (BiPAP) machine. Patient failed the BiPAP trial, was intubated and later extubated after five days, and discharged on room air. The patient was admitted to the intensive care unit due to toxic encephalopathy. Liver enzymes were markedly elevated during admission and trended down after supportive management, Narcan, and N-acetylcysteine treatment.

## Introduction

Drug-induced acute liver injury accounts for nearly 50% of acute liver failure [1]. Opioids and cocaine are the second and third most commonly abused drugs after cannabis [2]. Opioids are not usually implicated in a large series of cases of acute liver injury, but they have been shown to produce liver injury [2]. Cocaine affects the liver by causing hepatic necrosis, and liver enzymes trend after two days of usage. Liver impairment by cocaine is accompanied by other organ impairments, such as rhabdomyolysis, myocardial infarction, and renal failure. Cocaine causes liver necrosis noted by markedly elevated aminotransferases and lactate dehydrogenase (LDH) with minimal increase in alkaline phosphatase (ALP) [2]. Bilirubin increases two to three days later as compared to liver enzymes after acute liver injury. In some cases, the recovery is self-limited and the liver enzymes trend down within two weeks. N-acetylcysteine is usually given although it is not a specific treatment.

## Case presentation

We present the case of a 52-year-old male with a medical history of hypertension, asthma, chronic low back pain, hypothyroidism, chronic smoking, polysubstance abuse brought to the ER by emergency medical services (EMS). According to EMS, the patient was found unconscious at home with oxygen saturation (SpO2) of 86% on room air. The patient received Narcan 2 mg intranasally three times for suspected drug overdose and oxygen via nasal cannula. The patient became aroused to deep tactile and painful stimuli but was still drowsy. In the ER, the patient received IV Narcan and his mental status was improved. The patient was taking methadone 60 mg confirmed with methadone maintenance therapy program (MMTP). The history was limited at the time of presentation because of the altered mental status. The patient was noted to have labored breathing with the use of accessory respiratory muscle. His vital signs were as follows: blood pressure was 141/87 mmHg, pulse rate was 55 beats/minute, and respiratory rate was 35 breaths/minute. The patient was initially placed on a non-rebreather mask, then on a BiPAP since his oxygen saturation was not improving, and labored breathing continued.

The initial laboratory investigations on admission are depicted in Table 1. Liver enzymes were trended, peaked on day one as shown in Table 2. The coagulation profile was deranged, peaked on day one depicted in Table 3.

**Table 1:** Laboratory investigations on admission.

Investigation	Result	Normal range
Glucose	82	80-115 mg/dL
Potassium	6	4.5-5.5 mmol/L
Sodium	139	135-145 mmol/L
Anion gap	8	8-16 mmol/L
Blood urea nitrogen	13.7	8.4-25.7 mg/dL
Creatinine	2.02	0.72-1.25 mg/dL
Calcium	8.8	8.8-10.0 mg/dL
Albumin	3.4	3.4-5.4 g/dL
Magnesium	1.9	1.7-2.2 mg/dL
Phosphorus	2.5	2.5-4.5 mg/dL
Lactic acid	1.6	0.5-1.9 mg/dL
Creatinine phosphokinase	607	10-120 mcg/L
High sensitivity troponin	48.4	0.0-35.0 ng/L
B-natriuretic peptide	199	10-100 pg/mL

**Table 2:** Liver function test (LFT) trend. SGOT: serum glutamic oxaloacetic transaminase; SGPT: serum glutamic pyruvic transaminase

LFT trend	Alanine aminotransferase (ALT/SGPT)	Aspartate aminotransferase (AST/SGOT)	Alkaline phosphatase (ALP)	Total bilirubin
Normal value	10-55 U/L	5-34 U/L	40-150 U/L	0.2-1.2 mg/dL
On admission	873	712	63	0.8
Day 1	2548	3121	53	0.7
Day 2	2004	1234	52	0.9
Day 3	1614	848	59	1.4
Day 4	1507	433	54	1.5
Day 5	1342	315	58	1.6

**Table 3:** Coagulation profile trend. PT: prothrombin time; APTT: activated partial thromboplastin clotting time; INR: international normalized ratio

Coagulation profile trend	PT	INR	APTT
Normal value	11-13.5 seconds	<1.1	30-40 seconds
On admission	16.7	1.35	32.7
Day 1	17.0	1.61	34.9
Day 2	15.2	1.23	29.6
Day 3	13.9	1.12	32.9

Arterial blood gas revealed slight acidemia with pH 7.30, PO2 58.1 mmHg, PCO2 54.1 mmHg, and HCO3 26 mEq/L. Urine tested positive for opioids, cocaine, cannabis, and a prescription opioid, i.e., methadone. There were no urinary proteins. The hepatitis panel was negative. Cytomegalovirus (CMV) DNA polymerase chain reaction (PCR) was negative. Epstein-Barr virus (EBV) capsid Ag IgG Ab was positive at 400 U/mL, however, EBV capsid Ag IgM Ab was negative at <36 U/mL; EBV nuclear Ag IgG Ab was positive at >600 U/mL. The patient had a medical history of EBV infection. Herpes simplex virus (HSV) 1 and 2 IgM Ab were negative at <0.91, however, HSV 1 IgG Ab was positive at 16.50 and HSV 2 IgG Ab was positive at 20.0. HIV was negative. Viral hepatitis was ruled out. Iron profile and ferritin were normal. Serum ceruloplasmin was normal. Hemochromatosis and Wilson’s disease were unlikely. Shock liver was unlikely as the patient was not hypotensive during the entire admission. There was no alcohol history as per the patient and medical records, so alcoholic hepatitis was ruled out.

Chest x-ray and CT chest were normal. No record of alcohol history. Abdominal sonogram detected fatty liver. EKG showed normal sinus rhythm with VR 82 beats per minute (bpm), QTc 418 ms, with right axis deviation. The patient was managed in the intensive care unit for toxic encephalopathy. The patient was diagnosed with toxic encephalopathy and hepatotoxicity due to mixed drug overdose. He was treated with N-acetylcysteine as per protocol for poison control.

The rhabdomyolysis was indicated by increased creatinine phosphokinase (CPK) and adequately managed with fluid resuscitation. The initial increase in troponin was likely due to demand ischemia, which down trended after supportive management. Hyperkalemia was treated with insulin and dextrose. Acute renal failure was managed supportively. Liver enzymes were trending down after the last dose of N-acetylcysteine and the patient began to improve. He was observed for 24 hours in the general medical ward. He restarted on methadone maintenance therapy. Statin was not resumed until liver function was normalized, and the patient was asked to follow up with his primary care physician within two weeks of discharge.

## Discussion

Drug-induced acute liver failure is characterized by the emergence of symptoms of liver damage and encephalopathy in patients who do not have chronic liver disease. Acute liver failure often occurs within the first few months after starting a medication, but rarely takes longer than six months [2]. Treatment is mainly supportive and involves stopping the offending agents. In some cases, N-acetylcysteine can be used.

Opioid-induced liver injury or multi-organ failure is rare and usually not seen in everyday practice. In their study, Marks and Chapple found that transient liver damage is more often the result of a direct hepatotoxic effect of the large doses of heroin and cocaine used [3]. They reported that 80% of mixed heroin and cocaine users have alanine aminotransferase (ALT) elevation, and marked elevation occurs with relapse in cocaine and heroin use. Cocaine causes liver injury by conversion to a toxic metabolite as a result of P450 metabolism [3]. There have also been cases of heroin-induced pulmonary edema [4], acute cardiac injury [5], and acute rhabdomyolysis [6]. The intravenous form of buprenorphine has been linked to acute liver injury [7]. Moreover, unlike prescription opioids, the chronic use of heroin is strongly associated with liver fibrosis [8]. The use of hepatotoxic drugs combined with opioids increases the risk of liver injury. Cocaine has a short half-life, of about one hour, but its toxic metabolites may cause delayed coronary vasospasm and vasoconstriction to various organs [9]. Cocaine also activates the platelets and induces thrombus formation. N-acetylcysteine has been used in cocaine-induced hepatotoxicity due to its similar mechanism of acetaminophen-induced liver toxicity [10]. In our case, the liver enzymes trended down after a few days of drug wash-out and treatment with N-acetylcysteine and naloxone, which suggests that the hepatotoxicity was related to the patient’s multiple substance abuse, including heroin and cocaine. Multiple organ failure has been reported to be caused by cocaine but the risk is higher with the combined use of opioids and cocaine as reported by Marks and Chapple [3]. There are only a small number of literature on this subject and this case report will be an appropriate addition to it.

## Conclusions

Patients with a history of drug abuse especially cocaine and opioid users should be screened for liver failure. We suggest that drug-induced liver failure be considered as a differential diagnosis in all acute liver failure cases. Offending agents should be promptly discontinued. Cocaine and opioid overdose can become life-threatening. Therefore, patients should be followed up in outpatient settings for ongoing counseling and behavior modification.
